# Determination of Multiple Mycotoxins and Their Natural Occurrence in Edible Vegetable Oils Using Liquid Chromatography–Tandem Mass Spectrometry

**DOI:** 10.3390/foods10112795

**Published:** 2021-11-13

**Authors:** Thammaporn Junsai, Saranya Poapolathep, Samak Sutjarit, Mario Giorgi, Zhaowei Zhang, Antonio Francesco Logrieco, Peiwu Li, Amnart Poapolathep

**Affiliations:** 1 Department of Pharmacology, Faculty of Veterinary Medicine, Kasetsart University, Bangkok 10900, Thailand; thammaporn.ju@ku.th (T.J.); fvetsys@ku.ac.th (S.P.); 2 Faculty of Veterinary Technology, Kasetsart University, Bangkok 10900, Thailand; cvtsms@ku.ac.th; 3 Department of Veterinary Science, University of Pisa, 56122 Pisa, Italy; mario.giorgi@unipi.it; 4 Oil Crops Research Institute of the Chinese Academy of Agricultural Sciences, Wuhan 430062, China; zwzhang@whu.edu.cn (Z.Z.); peiwuli@oilcrops.cn (P.L.); 5 Institute of Sciences of Food Production, National Research Council, 70126 Bari, Italy; Antonio.logrieco@ispa.cnr.it

**Keywords:** mycotoxins, vegetable oils, QuEChERS, liquid chromatography-tandem mass spectrometry

## Abstract

The prevalence of mycotoxins is often increased by the climatic conditions prevailing in tropical regions. Reports have revealed the contamination of mycotoxins in some types of vegetable oil. However, vegetable oil is one of the essential ingredients used in food preparation. Thus, this study determined the occurrence of multi-mycotoxins in six types of vegetable oils commercially available in Thailand to assess the consumer health risk. In total, 300 vegetable oil samples (olive oil, palm oil, soybean oil, corn oil, sunflower oil, and rice bran oil) collected from various markets in Thailand were analyzed for the presence of nine mycotoxins, namely, aflatoxin B1 (AFB1), aflatoxin B2 (AFB2), aflatoxin G1 (AFG1), aflatoxin G2 (AFG2), beauvericin (BEA), ochratoxin A (OTA), zearalenone (ZEA), fumonisin B1 (FB1), and fumonisin B2 (FB2) using a quick, easy, cheap, effective, rugged, and safe (QuEChERS)-based procedure and a triple quadrupole mass spectrometer equipped with an electrospray ionization source. The incidences of mycotoxin contamination varied among the different types of oil samples. AFB1, AFB2, ZEA, FB1, and FB2 were most frequently found in contaminated samples. AFB2, BEA, ZEA, FB1, and FB2 contaminated olive oil samples, whereas AFB1, AFB2, AFG2, and OTA contaminated palm oil samples. AFB1, AFB2, and ZEA were found in soybean oils, whereas ZEA, FB1, and FB2 contaminated corn oil samples. AFB1 and AFG1 contaminated sunflower oil samples, whereas AFB1, AFB2, AFG1, and OTA were detected in rice bran oil samples. However, the contamination levels of the analyzed mycotoxins were below the regulatory limits.

## 1. Introduction

Mycotoxins are natural secondary metabolites produced by filamentous fungi, such as *Aspergillus, Penicillium*, and *Fusarium* genera [1]. Agricultural products can be contaminated starting from plant development in the field, through harvesting, and during the processing, storage, and transport of the final product [2]. Mycotoxin contaminations affect human health and economic factors [3]. To date, more than 400 mycotoxins with different levels of toxicity have been identified in cereals, fruits, vegetables, and other agricultural commodities [4]. Environmental changes and temperature fluctuations are the main factors affecting fungal growth and mycotoxin production [5]. Mycotoxin contamination can occur in a large variety of agricultural products, especially in seeds and grains, which are considered some of the most important staple foods globally. Mycotoxins are very persistent in food and are usually not completely eliminated during food processing operations [6]. Human health risks are usually associated with the direct consumption of food products contaminated with mycotoxins. The International Agency for Research on Cancer (IARC) classified AFB1 as a human carcinogen belonging to Group 1, with the formation of DNA adducts [7].

Oil is an important ingredient used in the preparation of foods [8], with edible oils being processed from various grains, such as soybean, rapeseed, sunflower seed, peanut, and olive [9]. The processed edible oils can easily become contaminated by mycotoxins originating in the seed before the crop has been harvested to produce their oils [10]. Oil from olives (*Olea europaea* L.) is a major product of the west coast USA, Turkey, Spain, Greece, Italy, and Morocco. This oil is considered to be a vital component of the human diet and is consumed along with bread or as an ingredient in salad and baked foods [4]. Contamination by aflatoxins (AFs) and ochratoxin A (OTA) is common in olive oil [4]. Hidalgo-Ruiz et al. [10] reported that 153 olive oil samples in Spain were contaminated by zearalenone (ZEA; 0.6–21.1 µg/kg), aflatoxin G1 (AFG1; 1.1–6.8 µg/kg), and aflatoxin G2 (AFG2; 0.8–1.9 µg/kg). Soybeans are a major source of protein for both human and animal consumption and can be contaminated by AFs, trichothecene (such as T-2 toxin), and cytochalasins [4]). Sunflower seeds (*Helianthus annuus* L.) are a good source of fiber, minerals, and vitamin E and have good antimicrobial and antioxidant properties, but the seeds can be contaminated by many fungi, such as *Fusarium verticillioides*, *Penicillium chrysogenum, Alternaria alternata, Aspergillus* spp., *Cladosporium* spp., *Drechslera* spp., and *Curvularia* spp. [4]. Maize (*Zea mays* L.) is one of the most important cereals globally, which is used as a basic raw material to prepare food products such as flour, grits, breakfast cereals, baby food, and germ oil; however, there have been many reports that it was contaminated by ZEA and fumonisin B1 (FB1) [4,11,12]. Rice bran oil is extracted from the inner husk and germ of rice (*Oryza sativa* L.). This oil is gaining popularity for its potential health benefits, as it contains ample amounts of oleic, linoleic, and palmitic acids. Again, there have been reports of various types of fungi in rice bran oil, such as *A. niger, A. flavus*, *A. candidus*, *A. glaucus*, *A. nidulans*, *A. fumigatus*, *Penicillium spp*., and *Gliocladium viride* [4]. The European Union (EU) mandated the maximum level of ZEA in refined maize oil at 400 µg/kg [13]. In addition, the EU set the maximum level in oilseed at 2.0 µg/kg for aflatoxin B1 (AFB1) and 4.0 µg/kg for the sum of four aflatoxins (AFB1, AFB2, AFG1, and AFG2) [14]. Considering the health impact of mycotoxin consumption, there is an urgent need to obtain data on multi-mycotoxin contamination in vegetable oils to better respond to potential problems. Therefore, the current study used a validated liquid chromatography- tandem mass spectrometry (LC–MS/MS) with a QuEChERS procedure to investigate the occurrence of multi-class mycotoxins, namely, AFB1, AFB2, AFG1, AFG2, OTA, ZEA, FB1, FB2, and beauvericin (BEA), in six types of common vegetable oils, namely, olive oil, palm oil, soybean oil, corn oil, sunflower oil, and rice bran oil, that have been commercialized in Thailand.

## 2. Materials and Methods

### 2.1. Reagents and Materials

The pure analytical standards (purity > 99.8%) of the mycotoxins AFB1, AFB2, AFG1, AFG2, OTA, and BEA were purchased from Romer Labs (Tulln, Austria), while ZEA, FB1, and FB2 were sourced from Sigma-Aldrich (Taufkirchen, Germany). Octadecyl (C18) was purchased from Macherey-Nagel (Düren, Germany). Analytical-grade sodium chloride and sodium phosphate were purchased from AppliChem GmbH (Darmstadt, Germany). Other reagents and chemicals were of analytical grade. Deionized distilled water was produced using the Milli-Q purification system from Millipore, Inc. (Bedford, MA, USA). Mycotoxins were dissolved in acetonitrile (ACN) and stored at −20 °C. Combinations of the nine standard mycotoxin stocks were prepared in acetonitrile at a concentration of 10 μg/mL.

### 2.2. Samples

In total, 300 samples of six different types of vegetable oils (olive oil, soybean oil, palm oil, maize oil, sunflower oil, and rice bran oil), using 50 samples per each type of vegetable oil, were randomly picked up from markets in Bangkok, Thailand. Different brands were selected to obtain a representative sample of products distributed in markets.

### 2.3. Sample Preparation

The QuEChERS procedure was modified from [8,10]. Briefly, 2 g of oil sample was added to 2 mL of Milli-Q water and mixed for 1 min using a vortex mixer. Then, 8 mL of the extraction solvent was added to a solution of 0.1% formic acid in 95% acetonitrile and mixed in a rotatory agitator for 30 min. Then, 4.0 g of Na_2_SO_4_ anhydrous salt and 1.00 g of NaCl were added into the mixture and shaken for 2–3 min. The ACN fraction was separated using centrifugation at 1968× *g* for 15 min. A sample of 7 mL of the supernatant fractions was transferred into a tube containing 200 mg of C18 and shaken for 2–3 min. The mixture was separated using centrifugation at 1968× *g* for 10 min. Then, 4 mL of the supernatant was evaporated until dry under a nitrogen stream at 50 °C on a digital heating device. The mobile phase solution was used to reconstitute the residue and injected into a 0.22 μm syringed filter (Sartorius AG, Goettingen, Germany) before being analyzed using LC–MS/MS.

### 2.4. LC–MS/MS Analysis

An LC–MS/MS system was used to determine contamination by the nine mycotoxins in the six different types of vegetable oils. The LC–MS/MS method was modified from Sinphithakkul et al. [15]. The chromatographic separation was performed on a ZORBAX Eclipse plus RRHD C18 column (50 × 4.6 mm, 1.8 μm particle size). The column was maintained at 40 °C. The mobile phase consisted of 5 mM ammonium formate with 0.2% formic acid in water (mobile phase A) and acetonitrile (mobile phase B) with a gradient elution: 0–1 min, 10% B; 1–5 min, 10–95% B; 5–10 min, 95% B; and 10–11 min, 10% B. The re-equilibration time for the analytical column was 4 min. The mobile phase solution was filtered through a 0.22 μm membrane (Sartorius AG, Goettingen, Germany) and ultrasonically degassed prior to application. The injection volume was 10 the while μL flow rate was 0.4 mL/min. The assay time was 15 min. A triple quadrupole mass spectrometer (6460 triple, Agilent Technologies, Waldbronn, Germany) equipped with an electrospray ionization source running in both positive and negative ion modes using the multiple reaction monitoring mode (MRM) was used for the analysis. The ionization source parameters were optimized as follows: capillary voltage, 3500 V; gas temperature, 320 °C; gas flow rate, 10 L/min; nebulizer, 50 psi. The parameters for mass spectrometry were optimized as displayed in Table 1.

### 2.5. Method Validation

Validation of the analytical method for AFB1, AFB2, AFG1, AFG2, OTA, ZEA, BEA, FB1, and FB2 was carried out to evaluate the efficiency of the present analytical method by investigating the repeatability, recovery, linear working range, limit of quantification (LOQ), limit of detection (LOD), precision, accuracy, and matrix effects, in accordance with the guidelines of SANTE/11813/2017 standard [16]. Representative matrices from each category were used to check the suitability of the proposed method for the determination of mycotoxins in the six different vegetable oils, linearity, limit of detection (LOD) and limit of quantification (LOQ), and precision. The LOD and LOQ of the method were evaluated as signal versus noise (S/N) values of 3:1 and 10:1, respectively.

### 2.6. Matrix Effects Study

The matrix effects of the method were evaluated using six types of extracts of blank matrix: olive oil, palm oil, soybean oil, corn oil, sunflower oil, and rice bran oil. Matrix-matched standards were prepared at seven levels in the range 0.1–500.0 ng/g by adding standard solution into the sample extract. The matrix effects expressing the matrix-induced SSE% were defined as percentage ratios of the matrix-matched calibration slope to the solvent calibration slope.

## 3. Results

### 3.1. Method Validation

The results of linearity and sensitivity are reported in Table 2, indicating that the method produced good linearity.

**Figure 1:**
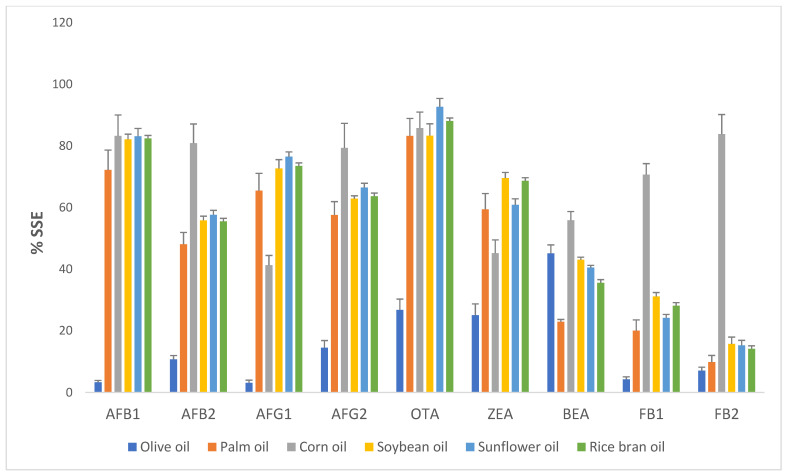
Signal suppression enhancement (%SSE) for nine mycotoxins in matrix-matched calibration.

All the method validation parameters in this study were sound: the correlation coefficients (R^2^) were not lower than 0.990, and the LOD and LOQ values ranged from 0.02 to 14.66 and 0.10 to 54.56 µg/kg, respectively (Table 2). The recovery and precision values met the acceptance criteria in the range of 70–120%, and the percentage relative standard deviation (%RSD) values were less than 20% [16] for all nine mycotoxins, as summarized in Table 3 for the samples of palm oil, corn oil, olive oil, sunflower oil, soybean oil, and rice bran oil, respectively. For identification requirements, the relative ion ratio from sample extracts was lower than 30% for all nine mycotoxins [16]. The evaluation of the matrix effect was shown for signal suppression for nine mycotoxins in six types of edible vegetable oils (Figure 1).

### 3.2. Occurrence of Multiple Mycotoxins in Edible Vegetable Oils

The method described was applied to a monitoring survey of nine mycotoxins in six types of edible vegetable oils (corn oil, palm oil, soybean oil, rice bran oil, olive oil, and sunflower oil). The summary of investigated compounds quantified in a variety of vegetable oils is shown in Table 4. These results showed mycotoxin contamination with at least one mycotoxin in each positive sample. The incidences of mycotoxins contamination varied among the different types of edible vegetable oil samples. AFB1, AFB2, ZEA, FB1, and FB2 were the most common in the contaminated oil samples. AFB2 (1.11–2.32 μg/kg), BEA (0.23–0.92 μg/kg), ZEA (29.17–208.54 μg/kg), FB1 (17.25–57.79 μg/kg), and FB2 (13.25–71.42 μg/kg) were found in contaminated olive oil samples, whereas AFB1 (0.2–0.37 μg/kg), AFB2 (0.44–1.05 μg/kg), AFG2 (0.44–2.59 μg/kg), and OTA (0.42–0.48 μg/kg) contaminated palm oil samples. AFB1 (0.21–0.27 μg/kg), AFB2 (0.35–0.48 μg/kg), and ZEA (59.31 μg/kg) were found in soybean oils, whereas ZEA (49.16–69.13 μg/kg), FB1 (5.69–9.68 μg/kg), and FB2 (32.64–101.41 μg/kg) contaminated corn oil samples. AFB1 (0.13–0.15 μg/kg) and AFG1 (0.10–0.12 μg/kg) contaminated sunflower oil samples, whereas AFB1 (0.27–0.49 μg/kg), AFB2 (0.58 μg/kg), AFG1 (0.31–0.32 μg/kg), and OTA (0.54–0.55 μg/kg) were detected in rice bran oil samples. The ranges and mean concentrations of the contaminating mycotoxins are shown in Table 4. However, none of the mycotoxin levels in any of the edible vegetable oil samples exceeded the legal limit prescribed by EU regulations. However, these results were consistent with the levels of contamination by AFB1, AFG, AFG2, and OTA reported in olive oil in southern Italy [17,18,19,20]. AFB2 was detected in olive oils in Iran [21]. In Spain, ZEA, FB1, and FB2 were found in corn oils [12], whereas ZEA was detectable with the range 100–600 μg/kg in Germany [22,23]. AFs and ZEA were found in sunflower oils in China and Tanzania [11,24]. In Pakistan, AFB1 contamination was found in soybean, corn, sunflower, and olive oils from imported and local samples [25].

## 4. Conclusions

The results demonstrated the successful development of an LC–MS/MS method as an excellent tool for the simultaneous identification of nine mycotoxins in six types of edible vegetable oil samples (olive oil, palm oil, soybean oil, corn oil, sunflower oil, and rice bran oil). The developed method was successfully validated according to the SANTE/11813/2017 standard. The results showed that contamination by mycotoxins co-occurred most commonly in the edible vegetable oil samples. The lowest percentage of mycotoxin contamination was in the sunflower oil samples, whereas the largest level of positive contamination was detected in corn oil samples. The mycotoxin levels of ZEA (<400 μg/kg) and fumonisins (<1000 μg/kg) in the corn oil samples almost complied with the EU regulations; however, there are no existing reports for the other mycotoxins investigated in this study. Further studies with a larger sample size are warranted to confirm these as acceptable levels.

## Figures and Tables

**Table 1:** MS/MS parameter for determination of nine mycotoxins.

Analyte	Precursor Ion (*m*/*z*)	Product Ion (*m*/*z*)	Collision Energy (eV)	Fragmentor (V)	Polarity
AFB_1_	313.07 QPI ^a^	285.1 241.0	21 35	150 150	Positive
AFB_2_	315.09 QPI ^a^	287.1 259.0	25 29	160 160	Positive
AFG_1_	329.1 QPI ^a^	311 243.0	25 43	160 160	Positive
AFG_2_	331.08 QPI ^a^	313 245.0	25 29	180 180	Positive
OTA	404 QPI ^a^	192.9 102.1	48 80	130 130	Positive
ZEA	319.16 QPI ^a^	283 187.0	5 17	80 80	Positive
BEA	801.4 QPI ^a^	784.3 244.1	13 35	160 160	Positive
FB_1_	722.4 QPI ^a^	352.5 334.4	40 45	160 160	Positive
FB_2_	706.3 QPI ^a^	336.2 318.3	35 40	200 200	Positive

^a^ Qualitative product ion.

**Table 2:** Linearity ranges (R^2^), limit of detection (LOD), and limit of quantification (LOQ) of optimized LC–MS/MS method for simultaneous determination of nine mycotoxins.

Analyte	Palm Oils			Corn Oils			Olive Oils		
	LOD (µg/kg)	LOQ (µg/kg)	R^2^	LOD (µg/kg)	LOQ (µg/kg)	R^2^	LOD (µg/kg)	LOQ (µg/kg)	R^2^
AFB_1_	0.03	0.12	0.999	0.07	0.24	0.996	0.14	0.47	0.995
AFB_2_	0.1	0.35	0.998	0.15	0.52	0.998	0.32	1.06	0.998
AFG_1_	0.07	0.26	0.999	0.07	0.26	0.997	0.25	0.83	0.993
AFG_2_	0.12	0.42	0.999	0.23	0.78	0.997	0.28	0.96	0.999
OTA	0.11	0.38	0.999	0.1	0.35	0.999	0.28	0.93	0.998
ZEA	10.8	36.02	0.990	14.66	48.87	0.991	7.95	26.51	0.991
BEA	0.34	1.14	0.999	0.27	0.92	0.996	0.07	0.23	0.997
FB_1_	2.11	7.03	0.990	1.66	5.56	0.996	4.24	14.14	0.990
FB_2_	4.53	15.1	0.990	4.99	16.63	0.995	3.94	13.14	0.997
**Analyte**	**Sunflower Oils**			**Soybean Oils**			**Rice Bran Oils**		
	**LOD ** **(µg/kg)**	**LOQ ** **(µg/kg)**	**R^2^**	**LOD ** **(µg/kg)**	**LOQ ** **(µg/kg)**	**R^2^**	**LOD ** **(µg/kg)**	**LOQ ** **(µg/kg)**	**R^2^**
AFB_1_	0.03	0.11	0.999	0.06	0.2	0.999	0.03	0.11	0.999
AFB_2_	0.04	0.14	0.999	0.1	0.34	0.999	0.05	0.18	0.998
AFG_1_	0.02	0.07	0.999	0.12	0.41	0.999	0.03	0.1	0.999
AFG_2_	0.64	2.14	0.999	0.15	0.52	0.999	0.33	1.11	0.999
OTA	0.22	0.75	0.999	0.3	1.01	0.999	0.12	0.42	0.998
ZEA	10.5	35.02	0.997	16.36	54.56	0.990	14.45	48.50	0.990
BEA	0.2	0.67	0.998	0.13	0.44	0.996	0.11	0.38	0.998
FB_1_	2.38	7.96	0.990	4.79	15.99	0.990	1.85	6.17	0.990
FB_2_	2.03	6.78	0.990	2.37	7.91	0.990	2.7	9.02	0.990

**Table 3:** Accuracy and precision for nine mycotoxins determined using optimal LC–MS/MS conditions for six different vegetable oil samples.

Mycotoxin (µg/kg)	Palm Oils			Corn Oils		
	Recovery (%) (*n* = 5)	Intraday Precision (%RSD) (*n* = 5)	Interday Precision (%RSD) (*n* = 5)	Recovery (%) (*n* = 5)	Intraday Precision (%RSD) (*n* = 5)	Interday Precision (%RSD) (*n* = 5)
AFB_1_						
0.5	114.2	3.5	5.3	80.4	5.1	4.9
2.5	94.2	2.6	7.4	93.5	2.1	6.2
10	92.4	3.5	5.2	98.2	1.1	2.3
AFB_2_						
0.5	96	2	12.1	95.7	6.3	5.8
2.5	115	9.5	9.1	79.5	7.6	6.4
10	110.7	14.3	8.4	72.4	2.6	2.9
AFG_1_						
0.5	93	5	5.9	93.7	6.4	7.7
2.5	105.5	2.3	2.4	92.5	4.5	4.6
10	112.9	3	4.3	89.3	2.8	3.8
AFG_2_						
0.5	104.1	13.1	13.2	85.8	12	12
2.5	114.7	7.8	3.8	97	12.2	8.2
10	105.3	4	3.9	93.8	3	2.6
OTA						
1	92.5	4.5	5.9	102	3.7	7.9
5	105.4	4.9	3.4	105	6.8	3.7
25	102.7	1.8	2.4	98.5	3	5.8
ZEA						
75	99.3	6	4.4	92.6	7.8	9
150	91	2	3.6	99.8	5.4	6.4
350	93	1.4	4	104.5	2.6	5.5
BEA						
0.5	84.3	2.9	3.2	81.6	10.6	7.6
2.5	88.2	2.3	3.8	88.3	6.4	5.3
10	93.3	1.5	2.9	90.7	8.4	7.1
FB_1_						
10	85	2.3	6.3	84.6	7.7	5.3
50	93.7	2.6	5.5	86.7	7.5	6.8
150	107.4	3.8	4.8	96.6	5.9	4.4
FB_2_						
10	84.8	4.6	3.7	95.6	5	4.5
50	101.7	6.5	5.9	96.2	3.5	4.3
150	103.3	4.4	4.8	101.4	2.8	2
**Mycotoxin ** **(µg/kg)**	**Olive Oils**			**Sunflower Oils**		
	**Recovery (%) (*n* = 5)**	**Intraday Precision ** **(%RSD) (*n* = 5)**	**Interday Precision ** **(%RSD) (*n* = 5)**	**Recovery (%) (*n* = 5)**	**Intraday Precision ** **(%RSD) (*n* = 5)**	**Interday Precision ** **(%RSD) (*n* = 5)**
AFB_1_						
0.5	82.7	4.4	5.4	84.7	12.5	10.2
2.5	91.2	4.8	4.9	91.1	1.6	11.6
10	94.1	7.3	5.5	96.1	3.9	10.4
AFB_2_						
0.5	86.3	5.8	6.2	90.1	4.8	5.2
2.5	91.5	2.4	3.4	93.3	3.9	3.7
10	96.2	3.8	4.8	94.3	2.3	10.2
AFG_1_						
0.5	85	8.2	7.1	95.7	5.7	5
2.5	87.6	7.4	5.7	93.7	2.3	2.2
10	90.7	6.9	8.3	103.4	4.5	8.6
AFG_2_						
0.5	108.7	3.9	2.9	98.6	7.1	5.3
2.5	101.6	4.1	4	95.4	6.5	4.1
10	105.1	3.6	3.9	92.6	3.2	10.6
OTA						
1	82.2	2.6	3.8	79.1	7.8	10.5
5	88.4	3.8	6.9	85	5.3	4.5
25	92.9	4	4.1	89.3	1.4	2.8
ZEA						
75	95.4	3.4	8.4	104.4	4.6	7.1
150	100.1	2.1	2.5	92.4	2.7	3.6
350	104.3	2.2	2.7	92.6	5	4.7
BEA						
0.5	84.6	8	7.5	115.7	3.1	3.8
2.5	106.1	9.3	11.8	117.2	10.6	8.1
10	91.1	11	9.7	114.1	13.5	8.2
FB_1_						
10	106.6	6.1	4.7	84.2	1.8	2.3
50	93.2	4.3	4.5	92.6	2.1	4.1
150	90.6	3.3	3.6	95.1	4.6	2.6
FB_2_						
10	93.4	5.3	6.1	84	8.4	5.4
50	106.6	7.7	5.3	90.6	8.3	4.7
150	100.1	7.8	4.3	87.4	8.9	8.3
**Mycotoxin ** **(µg/kg)**	**Soybean Oils**			**Rice Bran Oils**		
	**Recovery (%) (*n* = 5)**	**Intraday Precision ** **(%RSD) (*n* = 5)**	**Interday Precision ** **(%RSD) (*n* = 5)**	**Recovery (%) (*n* = 5)**	**Intraday Precision ** **(%RSD) (*n* = 5)**	**Interday Precision ** **(%RSD) (*n* = 5)**
AFB_1_						
0.5	101.1	7.05	8.9	103.3	1.9	4.1
2.5	98.6	1.6	3.2	91.6	3.6	2.3
10	105.4	5.1	6.8	89.7	4.5	5.2
AFB_2_						
0.5	84.2	19.7	8.2	84.3	6.1	4.1
2.5	92.8	3.8	5.3	90.6	1.9	3.5
10	93.4	2.9	5.2	88.4	4.6	4.7
AFG_1_						
0.5	84.8	6.9	7.5	89.4	4.7	5.5
2.5	101.7	3.9	3.8	86.4	4	2.9
10	105.3	4.1	5.2	83.1	4.6	5.1
AFG_2_						
0.5	88.7	3.1	6.2	85.6	6.6	6.9
2.5	91.6	4.6	5.9	83.6	3.2	4.8
10	103.3	2.9	6.5	75.7	5.9	5.6
OTA						
1	81.4	10.7	10.4	93.7	6.2	7.9
5	74.9	5.7	7.7	77.9	4.6	3.6
25	75.6	0.5	2.4	78	5.5	6.2
ZEA						
75	85.6	8.6	7.5	79.7	3.6	4.4
150	88.6	1.5	3.8	92.9	2.9	3.2
350	101.2	7.4	7.8	93.1	6.2	4.4
BEA						
0.5	85.6	6.6	7.4	87.1	10.3	8.5
2.5	89.1	6.3	7	85.6	8.2	7.3
10	86.6	8.7	10.3	88.2	5.8	6.4
FB_1_						
10	83.3	3.3	6.3	84.5	3.1	3.5
50	92.7	3.6	4.5	86	5.2	4.8
150	104.6	4.8	5.5	90.6	4.6	5.8
FB_2_						
10	82.3	3.6	4.1	86.8	4.6	5.1
50	99.7	5.6	6.9	103.7	6.2	6.9
150	103.3	4.4	4.9	106.3	6.3	7.4

%RSD = percentage relative standard deviation.

**Table 4:** Occurrence of nine mycotoxins in edible vegetable oils.

Mycotoxin	Palm Oils (*n* = 50)			Corn Oils (*n* = 50)			Olive Oils (*n* = 50)		
	No. of Positive Samples	Level (µg/kg)		No. of Positive Samples	Level (µg/kg)		No. of Positive Samples	Level (µg/kg)	
		Range	Mean ^a^		Range	Mean ^a^		Range	Mean ^a^
AFB_1_	27	0.20–0.37	0.25	0	<LOD	<LOD	0	<LOD	<LOD
AFB_2_	6	0.44–1.05	0.70	0	<LOD	<LOD	18	1.11–2.32	1.76
AFG_1_	0	<LOD ^b^	<LOD	0	<LOD	<LOD	0	<LOD	<LOD
AFG_2_	6	0.44–2.59	0.87	0	<LOD	<LOD	0	<LOD	<LOD
OTA	11	0.42–0.48	0.44	0	<LOD	<LOD	0	<LOD	<LOD
ZEA	0	<LOD	<LOD	5	49.16–69.13	60.33	12	29.17–208.54	116.33
BEA	0	<LOD	<LOD	0	<LOD	<LOD	20	0.23–0.92	0.30
FB_1_	0	<LOD	<LOD	8	5.69–9.68	7.27	2	17.25–57.79	37.52
FB_2_	0	<LOD	<LOD	42	32.64–101.41	62.55	11	13.25–71.42	24.27
**Mycotoxin**	**Sunflower Oils (*n* = 50)**			**Soybean Oils (*n* = 50)**			**Rice Bran Oils (*n* = 50)**		
	**No. of Positive Samples**	**Level ** **(µg/kg)**		**No. of Positive Samples**	**Level ** **(µg/kg)**		**No. of Positive Samples**	**Level ** **(µg/kg)**	
		**Range**	**Mean ^a^**		**Range**	**Mean ^a^**		**Range**	**Mean ^a^**
AFB_1_	4	0.13–0.15	0.14	20	0.21–0.27	0.22	26	0.27–0.49	0.31
AFB_2_	0	<LOD ^b^	<LOD	10	0.35–0.48	0.41	1	0.58	0.58
AFG_1_	22	0.10–0.12	0.10	0	<LOD	<LOD	5	0.31–0.32	0.31
AFG_2_	0	<LOD	<LOD	0	<LOD	<LOD	0	<LOD	<LOD
OTA	0	<LOD	<LOD	0	<LOD	<LOD	6	0.54–0.55	0.54
ZEA	0	<LOD	<LOD	1	59.31	59.31	0	<LOD	<LOD
BEA	0	<LOD	<LOD	0	<LOD	<LOD	0	<LOD	<LOD
FB_1_	0	<LOD	<LOD	0	<LOD	<LOD	0	<LOD	<LOD
FB_2_	0	<LOD	<LOD	0	<LOD	<LOD	0	<LOD	<LOD

^a^ Mean concentrations were calculated from positive samples. ^b^ LOD, limit of detection.
